# Using case-level context to classify cancer pathology reports

**DOI:** 10.1371/journal.pone.0232840

**Published:** 2020-05-12

**Authors:** Shang Gao, Mohammed Alawad, Noah Schaefferkoetter, Lynne Penberthy, Xiao-Cheng Wu, Eric B. Durbin, Linda Coyle, Arvind Ramanathan, Georgia Tourassi

**Affiliations:** 1 Computational Sciences and Engineering Division, Oak Ridge National Laboratory, Oak Ridge, TN, United States of America; 2 Division of Cancer Control and Population Sciences, National Cancer Institute, Bethesda, MD, United States of America; 3 Louisiana Tumor Registry, Louisiana State University Health Sciences Center School of Public Health, New Orleans, LA, United States of America; 4 Kentucky Cancer Registry, University of Kentucky, Lexington, KY, United States of America; 5 Information Management Services Inc, Calverton, MD, United States of America; 6 Data Science and Learning Division, Argonne National Laboratory, Lemont, IL, United States of America; The Jackson Laboratory for Genomic Medicine, UNITED STATES

## Abstract

Individual electronic health records (EHRs) and clinical reports are often part of a larger sequence—for example, a single patient may generate multiple reports over the trajectory of a disease. In applications such as cancer pathology reports, it is necessary not only to extract information from individual reports, but also to capture aggregate information regarding the entire cancer case based off case-level context from all reports in the sequence. In this paper, we introduce a simple modular add-on for capturing case-level context that is designed to be compatible with most existing deep learning architectures for text classification on individual reports. We test our approach on a corpus of 431,433 cancer pathology reports, and we show that incorporating case-level context significantly boosts classification accuracy across six classification tasks—site, subsite, laterality, histology, behavior, and grade. We expect that with minimal modifications, our add-on can be applied towards a wide range of other clinical text-based tasks.

## Introduction

Electronic health records (EHRs) are a prevalent and detailed source of health data—according to the Office of the National Coordinator for Health Information Technology, as of 2017, 86% of office-based physicians store health records electronically [1]. These EHRs record detailed information from all the clinicians involved in a patient’s care—this can include demographics, progress notes, medications, vital signs, past medical history, immunizations, laboratory tests and results, radiology reports, and more [2]. As a result, EHRs are an important tool for public health surveillance and for monitoring communicable and chronic diseases [3].

One notable property of EHRs is that they often come in a sequence—a single patient or case may generate multiple reports over time. Within the same sequence, EHRs are generally related to each other in some manner; for example, the diagnosis of a disease in one EHR may indicate additional tests for that disease in following EHRs, and later EHRs may document the treatment or progression of the disease. In some applications, for any given clinical report, it is helpful or necessary to extract aggregate information using other reports in the sequence [4–6]. An important example is cancer pathology reports—individual cancer pathology reports may need to be tagged with aggregate labels that describe the cancer case as a whole, and these aggregate labels require collective analysis of all pathology reports belonging to a given cancer case.

Because of the sequential nature of EHRs, existing work has explored how to predict clinical events, phenotype patients, and perform other medical tasks based off structured time-series data extracted from a patient’s EHRs. For example, Cheng et. al. used a convolutional neural network (CNN) based architecture on sequentially-ordered medical events (e.g., international classification of disease codes) extracted from EHRs to predict the onset of Congestive Heart Failure and Chronic Obstructive Pulmonary Disease [7], and Lipton et. al. used a recurrent neural network (RNN) based architecture on a time-series of clinical measurements (e.g., blood pressure and heart rate) extracted from EHRs to diagnose diseases [8]. Additional examples of deep learning approaches on sequential structured data extracted from EHRs are available in review papers [9, 10]. To our knowledge, existing research that utilizes sequential analysis of EHRs does not use raw natural language as input; rather, they utilize pre-extracted features from EHRs, such as diagnosis codes, medication codes, and procedure codes. As a result, relevant information in the form of natural language, such as those from clinical notes, is not captured by these approaches.

Existing work has also explored how to extract useful information from natural language in EHRs without incorporating any sequential context. For example, Mullenbach et. al. use a CNN-based architecture to extract medical event codes from individual clinical notes [11], and Jagannatha and Yu perform the same task utilizing an RNN-based architecture [12]. Recent work using Transformer-based architectures has also explored natural language inference and named entity recognition tasks on individual clinical notes [13]; notably, [14] utilizes limited sequential context in hospital readmission classification by concatenating multiple clinical notes from the same patient, but the final concatenated text is then split into short chunks and each chunk analyzed independently. A more comprehensive list of NLP approaches on individual clinical texts is available in review papers [9, 15, 16]. Because existing natural language processing (NLP) approaches for clinical text process each document independently from any others, any useful relationships between EHRs belonging to the same patient or case are not captured.

There is a small body of existing research on incorporating sentence-level context for general NLP tasks outside of the clinical domain. Dernencourt and Lee examined how sentence-level context could be used to improve classification of short text sequences in day-to-day dialog [17], and Jin and Szolovits used a similar approach to examine how sentence-level context could improve classification of individual sentences in biomedical and scientific abstracts [18]. The results from these works show that taking advantage of contextual information outside of the target sentence boosts the performance of certain tasks. We propose building upon these works and extending them to the domain of natural language in EHRs—we expect that performance in information extraction from unstructured clinical text can be improved by accounting for contextual information from related text, such as those from other EHRs belonging to the same patient or case.

In this paper, we present a simple modular add-on for capturing and utilizing sequential, case-level context that is designed to be compatible with most existing deep learning architectures for classifying individual documents. We focus on the task of classifying key data elements in sequences of cancer pathology reports; this is not only an essential task for cancer surveillance and for supporting further cancer research, but it is also highly labor-intensive and could greatly benefit from automation. Using this task, we test our modular add-on with two existing deep learning architectures—word-level CNNs [19], which are widely used across many EHR-based applications [20–23], and hierarchical self-attention networks (HiSANs) [24], the current state-of-the-art in cancer pathology report classification. We show that our add-on improves the effectiveness of both networks in classifying six key data elements that have been identified by the National Cancer Institute (NCI) Surveillance, Epidemiology, and End Results (SEER) program as essential for cancer surveillance—site, subsite, laterality, behavior, histology, and grade—using a corpus of approximately 430K cancer pathology reports. We expect that with minimal modifications, our add-on may improve performance across a wide range of other EHR- and clinical text-based tasks.

## Materials and methods

### Problem description

Suppose we have a sequence of *n* text-based EHRs (e.g., clinical notes) *d*_0_, *d*_1_, …, *d*_*n*_ which are ordered by the date the report was created. All reports in the sequence are related to each other—for example, all reports belong to the same patient or case. Each report is associated with a label *y*_*i*_, where *y*_*i*_ is the label for the *i*th report. The task is to predict the labels *y*_*i*_ for each document *d*_*i*_ in the sequence.

In the baseline case, which has been explored in previous research, a machine learning or deep learning model predicts the label *y*_*i*_ for *d*_*i*_ independently from any other reports in the sequence. In other words, *y*_*i*_ = Predict(*d*_*i*_). In this paper, we explore methods to incorporate contextual information from all reports in the sequence, such that *y*_*i*_ = ContextAwarePredict(*d*_*i*_|*d*_0_, …, *d*_*n*_).

To simulate applications in the real world, we apply restrictions based off two different scenarios. In the first scenario, when processing a report *d*_*i*_, all other reports in the sequence are available; the predictive model can utilize contextual information from other reports that came both before and after the target report. This first scenario represents offline applications using historical data where for any given patient/case, all EHRs for that patient/case are available.

In the second scenario, when processing a report *d*_*i*_, only reports that came before *d*_*i*_ are available; the predictive model can only utilize contextual information from reports that came before the target report such that *y*_*i*_ = ContextAwarePredict(*d*_*i*_|*d*_0_, …, *d*_*i*−1_). This second scenario represents online applications where EHRs must be immediately processed as they arrive and information from future reports does not yet exist.

### Capturing case-level context

**Fig 1:**
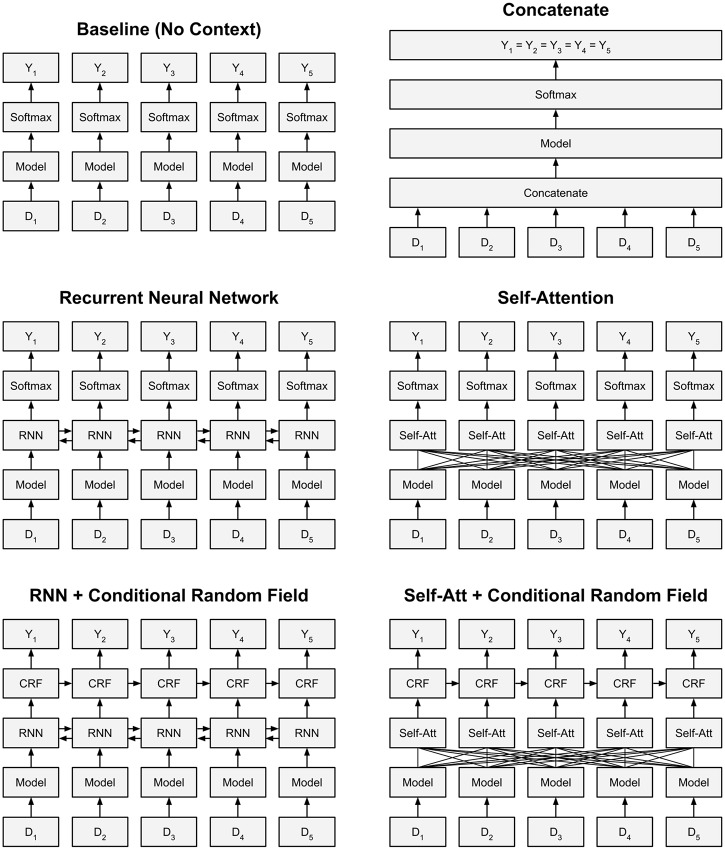
The baseline case for classifying EHRs and the five methods for incorporating case-level context from other reports. In the figures above, “Model” represents an arbitrary deep learning model designed for text classification, the output of which is an embedding representation of the input document.

We explore five different methods for incorporating case-level context when extracting information from text-based EHRs—concatenation, RNNs, RNNs with linear-chain conditional random field (CRF), self-attention, and self-attention with linear-chain CRF. These are described in greater detail in the following sections. Fig 1 illustrates the baseline case (without incorporating case-level context) and each of the five methods.

#### Concatenation

The most simple and naive way to incorporate case-level context is to concatenate all reports belonging to the same patient/case, as shown in Eq 1. Because the model has access to information from all reports in the sequence, it can utilize information from other reports for decision making on any given report.

$$\begin{array}{c} y_{i}=\text{Predict}\left(\left[d_{0},\ldots ,d_{i},\ldots ,d_{n}\right]\right) \end{array}$$ (1)

This strategy is only valid under the condition that all reports within a given sequence share the same label; that is, *y*_*i*_ = *y*_*j*_ for all *i* and *j* in the sequence. For example, in our application, all cancer pathology reports associated with the same unique tumor ID are tagged with the same aggregate-level labels. This strategy fails under the condition where each report in the sequence has a different label because the model would be forced to predict different labels from the same input.

Another notable limitation of concatenation is that it significantly increases the length of the input text that is fed into the model. Depending on the type of model used, this can cause severe problems. For example, RNN-based models are extremely slow and difficult to train when input sequences become too long [25, 26]; likewise, the memory required by self-attention-based models scales quadratically based off input length [27]. For long sequences where *n* is large, many models may become prohibitively expensive in terms of time and/or space complexity. In our experiments, we found that even the memory-efficient text CNN [19] has memory issues when the input sequence length exceeds 20K tokens, which was easily reached when concatenating sequences of 20+ pathology reports.

#### Recurrent neural networks

RNNs are a type of neural network architecture designed to process sequential information [28]. RNNs take in a series of inputs and produce a series of outputs. At any given timestep in the series, the output of the RNN depends not only on the input at the current timestep, but also on the inputs from all previous timesteps. This allows RNNs to recognize meaningful patterns over a sequence of entries, such as a series of EHRs over time.

The two most popular types of RNNs are long short-term memory (LSTMs) [29] and gated recurrent units (GRUs) [30]. Whereas more basic RNNs treat every entry in a sequence with equal importance, LSTMs and GRUs utilize gating operations to recognize when to save important information and when to skip less relevant entries; this allows LSTMs and GRUs to recognize more complex patterns over much longer sequences. In this work, we use GRUs because they have previously performed slightly better than LSTMs on EHRs and biomedical text [12, 31]. The operations for a GRU are shown below:

$$\begin{array}{c} \begin{array}{cc} z_{t} & =\sigma (W_{z}\left[h_{t-1},x_{t}\right]+b_{z})\\ r_{t} & =\sigma (W_{r}\left[h_{t-1},x_{t}\right]+b_{r})\\ c_{t} & =tanh(W_{c}\left[r\circ h_{t-1},x_{t}\right]+b_{c})\\ h_{t} & =\left(1-z_{t}\right)\circ h_{t-1}+z\circ c_{t} \end{array} \end{array}$$ (2)

In the equations above, *c*_*t*_ is the processed value of the current input, which is a combination of the current input *x*_*t*_ and previous output *h*_*t*−1_. *r*_*t*_ is a “reset gate” that controls the influence of the previous output *h*_*t*−1_ when calculating *c*_*t*_. Finally, *z*_*t*_ is an “update gate” that determines how to combine *c*_*t*_ with the previous output *h*_*t*−1_ to generate the final output at the current timestep. Each operation relies on a function based on a learned weight *W* and bias *b* and the concatenation of the output from the previous timestep *h*_*t*−1_ and the input at the current timestep *x*_*t*_.

To capture case-level context from EHRs, we utilize a GRU in conjunction with an existing deep learning text classification model designed to classify single reports, such as a text CNN [19]. Generally speaking, deep learning models designed for text classification will first encode a document into a final “document embedding”, which is then passed onto a softmax layer for classification. The document embedding is usually generated by the penultimate layer of the deep learning model, and it represents the most important information used to classify a given document. Given a sequence of EHRs *d*_0_, …*d*_*i*_, …, *d*_*n*_, we first use an existing deep learning model to generate document embeddings *e*_0_, …*e*_*i*_, …, *e*_*n*_ for each report. We then feed these into a GRU (with optional bidirectionality) as follows:

$$\begin{array}{c} \begin{array}{cc} o_{i} & =\text{BiGRU}(e_{0},\ldots ,e_{i},\ldots ,e_{n})\\ y_{i} & =\text{Softmax}(W_{s}o_{i}+b_{s}) \end{array} \end{array}$$ (3)

where *o*_*i*_ is the *i*th output generated by the GRU. *o*_*i*_ is then fed into a softmax classifier or linear-chain CRF to generate the final label *y*_*i*_. When making a decision for any given EHR, the GRU can take advantage of contextual information from other EHRs that came before (and in the case of bidirectionality, after) that report.

#### Self-attention

Self-attention is a relatively new alternative to RNNs made popular by the Transformer architecture [32]. Like RNNs, self-attention takes in a series of inputs and generates a series of outputs; however, self-attention has been shown to both achieve higher accuracy and run faster than RNNs on a wide range of NLP tasks [33–35]. In our work, we use an implementation similar to that from the original Transformer paper, which is described below:

$$\begin{array}{c} \begin{array}{c} Q=\text{ELU}(\text{Conv}1\text{D}\left(X+P,W_{q}\right)+b_{q})\\ K=\text{ELU}(\text{Conv}1\text{D}\left(X+P,W_{k}\right)+b_{k})\\ V=\text{ELU}(\text{Conv}1\text{D}\left(X+P,W_{v}\right)+b_{v})\\ \text{Self-Attention}\left(Q,K,V\right)=\text{softmax}\left(\frac{QK^{T}}{\sqrt{d}}\right)V \end{array} \end{array}$$ (4)

In the equations above, $X\in R^{n\times d}$ is a matrix of the entries in the input sequence, where *n* is the length of the sequence and *d* is the dimension size of each entry. $P\in R^{n\times d}$ are positional embeddings [36, 37] that represent the absolute position of each entry in the sequence—this simply allows the self-attention module to capture information about the order of the entries in the sequence. In our application, *P* is randomly initialized and learned through training. *X* + *P* is fed into three parallel 1D-convolution operations (with a window size of one entry and exponential linear unit activation [38]) to extract three different feature representations of the input sequence—*Q*, *K*, and *V*. *W*_*q*_, *W*_*k*_, *W*_*v*_, *b*_*q*_, *b*_*k*_, and *b*_*v*_ are the weights and biases associated with each 1D convolution. The dot product of *Q* and *K* forms a *n* × *n* similarity matrix which captures the relationships between each entry in the sequence. The final output is a new sequence $O\in R^{n\times d}$ in which each entry has captured information from all entries in the original sequence related to that entry.

For our implementation, we also utilize the multihead variant of self-attention, which splits the self-attention operation into *h* parallel sub-attention operations. The inputs into self-attention are split across the *d* dimension such that $\left\{Q_{i},K_{i},V_{i},\right\}\in R^{n\times d/h}$; this enables each sub-attention to focus on a different portion of the feature space and has been shown to give a slight boost to performance [32]:

$$\begin{array}{c} \begin{array}{c} \text{Multihead}\;\text{Self-Att}\left(Q,K,V\right)=\left[h_{1},\ldots ,h_{h}\right]\\ \text{where}\;h_{i}=\text{Self-Attention}\left(Q_{i},K_{i},V_{i}\right) \end{array} \end{array}$$ (5)

Like in the case of RNNs, to capture case-level context from EHRs, we use self-attention in conjunction with an existing deep learning architecture for text classification. Given a sequence of EHRs *d*_0_, …*d*_*i*_, …, *d*_*n*_, we first use an existing deep learning model to generate document embeddings *e*_0_, …*e*_*i*_, …, *e*_*n*_ for each report. This creates the input matrix $E\in R^{n\times d}$, which takes the place of *X* in Eqs 4 and 5; the self-attention operations then allow for capture of contextual information from other EHRs in the sequence. The output from self-attention is fed into a final softmax layer or linear-chain CRF for classification.

#### Softmax vs. linear-chain conditional random field

Our RNN and self-attention methods can utilize either a softmax or linear-chain CRF as the final layer for label generation. Incorporating a linear-chain CRF instead of a softmax after an RNN has previously been shown to improve performance on various general NLP sequence tagging tasks, such as in part-of-speech tagging and named entity recognition [39].

$$\begin{array}{c} P\left(y_{i}\right)=\frac{\text{exp}(W_{s}o_{i}+b_{s})}{\sum \text{exp}(W_{s}o_{i}+b_{s})} \end{array}$$ (6)

We use the standard implementation of softmax for our softmax layer, which is described in Eq 6. *y*_*i*_ is the label associated the *i*th report in a sequence, *o*_*i*_ is the RNN or self-attention output associated the *i*th report in a sequence, and *W*_*s*_ and *b*_*s*_ are the learned weight and bias parameters.

$$\begin{array}{c} \begin{array}{c} P\left(\bar{y}\right)=\frac{\text{exp}(W_{c}F\left(\bar{o},\bar{y}\right))}{\sum \text{exp}(W_{c}F\left(\bar{o},\bar{y}\right))}\\ \text{where}\;F\left(\bar{o},\bar{y}\right)=F\left(y_{i-1},y_{i},\bar{o},i\right) \end{array} \end{array}$$ (7)

We use the standard implementation of a linear-chain CRF layer for our CRF layer, which is described in Eq 7. *y*_*i*_ is the label associated the *i*th report in a sequence, $\bar{y}$ is all labels associated with the sequence, *o*_*i*_ is the RNN or self-attention output associated the *i*th report in a sequence, $\bar{o}$ is all outputs associated with the sequence, and *W*_*c*_ are the learned weight parameters.

Compared to softmax, the main difference is that the linear-chain CRF utilizes a feature function $F(\bar{o},\bar{y})$ rather than directly utilizing *o*_*i*_. When predicting *y*_*i*_, this feature function not only utilizes *o*_*i*_ to identify the correct label for *y*_*i*_ but also incorporates the transition probabilities between consecutive labels *y*_*i*_ and *y*_*i*−1_ in a sequence. For example, in our specific application of cancer pathology reports, all reports within the same sequence are tagged with the same labels; therefore, the CRF should learn that given the label *y*_*i*−1_ of the previous entry, the probability of *y*_*i*_ transitioning to a different label is extremely low.

#### Modular vs. end-to-end training

Except for the concatenation method, all other methods to capture case-level context are modular in that they can be trained independently from an existing deep learning model for text classification in a two-step fashion. A user can choose an existing deep learning text classification model designed to classify single documents, train it on a corpus of EHR texts, and use the trained model to generate document embeddings for each EHR; then, the user can train our case-level context module (e.g., RNN or self-attention with or without CRF) independently on the resulting document embeddings. The benefit of modular training is that it eliminates the necessity of engineering the RNN/self-attention/CRF layers directly into an existing model architecture, which may potentially create overly cumbersome models that are computationally burdensome.

If desired, the RNN/self-attention/CRF layers can still be integrated directly into an existing text classification model such that training is end-to-end. We compare the performance of modular two-step training with end-to-end training using text CNNs and show that training the RNN, self-attention, and CRF layers in a modular fashion results in similar performance compared to end-to-end training.

### Dataset

As part of the national cancer surveillance mandate, the SEER cancer registries collect data on patient demographics, primary tumor site, tumor morphology, stage at diagnosis, and first course of treatment. Tumor site and morphology are captured in the form of six key data elements—site, subsite, laterality, histology, behavior, and grade. These data elements are considered essential for SEER to provide an annual report on cancer incidence.

Our full dataset consists of 546,806 cancer pathology reports obtained from the Louisiana and Kentucky SEER cancer registries. Data was utilized under a protocol approved by the Department of Energy Central IRB. For our study, we use original pathology reports that did not go through de-identification; this study qualified for a waiver of subject consent according to 10 CFR 745.117(c).

Our dataset covers cancer cases of all types from Louisiana residents spanning the years 2004-2018 and Kentucky residents spanning the years 2009-2018. Each pathology report is associated with a unique tumor ID that indicates the specific patient and tumor for the report—each tumor ID may be associated with one or more pathology reports. For example, a patient may have an initial test to check for cancer at a particular site, secondary tests of neighboring organs to see if the cancer has spread, and a followup test to see if the cancer has developed.

Each unique tumor ID is tagged with aggregate ground truth labels for six key data elements—site, subsite, laterality, histology, behavior, and grade. These ground truth labels were manually annotated by a human expert with access to all data relevant to each tumor ID; this includes radiology reports and other clinical notes not available in our dataset. The SEER cancer registries require that each individual cancer pathology report be labelled with the aggregate tags belonging to its associated tumor ID. Therefore, all pathology reports associated with the same tumor ID will have the same labels. Each pathology report is labeled with one of 70 possible sites, 314 possible subsites, 7 possible lateralities, 4 possible behaviors, 547 possible histologies, and 9 possible grades; a detailed breakdown of number of instances per label is available in S1 Fig of our supporting information. A notable challenge in automated classification of cancer pathology reports, which is captured by our dataset, is identifying the correct aggregate-level labels for each report in a tumor ID sequence, even if some reports are addenda that may not contain the necessary information for all six data elements.

A large number of cancer pathology reports in our dataset are associated with tumor IDs that have only a single pathology report; in other words, these pathology reports do not have any case-level context because there is only a single report in the sequence. Because these reports do not require case-level context for analysis, they are filtered out of our dataset. After filtering, our dataset consists of 431,433 pathology reports and 135,436 unique tumor IDs; on average, each tumor ID is associated with 3.2 pathology reports. A more detailed histogram of the number of reports per tumor ID is available in S2 Fig of our supporting information.

To simulate a production setting in which a model trained on older, existing reports must make predictions on new incoming data, we split our dataset into train, validation, and test sets based off date. We first group pathology reports by tumor ID. If any tumor ID is associated with a report dated 2016 or later, all reports from that tumor ID are placed in our test set. On the remaining reports, we use 80:20 random splitting to create our train and validation sets, ensuring that reports from the same tumor ID are all placed in the train set or in the validation set without being split between the two. This yields a train set of 258,361 reports, a validation set of 64,906 reports, and a test set of 108,166 reports. Due to the long training time associated with deep learning models, cross validation is not used.

We apply standard text preprocessing techniques including lowercasing text, replacing hex and unicode, and replacing unique words appearing fewer than five times across the entire corpus with an “unknown_word” token. A more detailed description of our text cleaning process is available in our supporting information.

### Baseline models

To capture case-level context, our RNN-based and self-attention-based approaches work in conjunction with an existing deep learning text classification model, which is used to produce the document embeddings for individual pathology reports. For this study, we utilize two deep learning text classification models that have previously been shown to be highly effective for classifying cancer pathology reports—a CNN [40, 41] and a HiSAN [24].

The CNN is an adaptation of the common word-level CNN used for general NLP tasks [19]—it examines combinations of three, four, and five consecutive words at a time and identifies the most salient word combinations for a given task. The HiSAN is a newer approach that utilizes a hierarchical structure based off self-attention to identify meaningful combinations of words in a document; compared to the CNN, the HiSAN can capture longer-distance word relationships that may be useful for a given task. To our knowledge, the HiSAN is the current state-of-the-art in cancer pathology report classification. Because the CNN and HiSAN were both developed on a similar dataset to ours, we use the exact same architecture and hyperparameter settings as those described in the original publications; for additional details, we refer the reader to the original papers.

## Experiments

### Setup details

Our experiments are designed to compare the performance of our five proposed methods to capture report level context under different scenarios. For each of these five methods, we test using both the CNN and the HiSAN as the baseline approaches. For the all methods other than concatenation, the CNN and HiSAN are first trained independently on the individual reports in our corpus (without case-level context), and then the resulting document embeddings are saved and used as input. We test performance on six classification tasks on our corpus—site, subsite, laterality, histology, behavior, and grade.

As described in our problem description, we test our methods under two conditions. In the first, for any given pathology report in a sequence of reports, each method can access other reports that came both before and after that report. In the second, each method can only access other reports that came before that report. For the concatenation method, this is achieved by concatenating only content from reports that came before the target report. For the RNN-based method (with and without CRF), we use a unidirectional RNN that can only access information from previous entries rather than a bidirectional RNN that can see both forward and backward. In the self-attention-based method (with and without CRF), we add a masking layer such that for any given entry in the sequence, self-attention will only find relationships between that entry and previous entries in the sequence.

We tune the hyperparameters of our RNN-based method and self-attention-based method using our validation set. For the RNN-based method, we use a GRU with hidden size 300, and for the self-attention based method, we use multihead self-attention with 300 dimensions and 6 heads. As we noted previously, concatenation can be prohibitively expensive for more complex models because the input documents can become very long. Therefore, we test the concatenation method using the CNN baseline model only, as the HiSAN was unable to fit the concatenated documents into memory.

Except for concatenation, our approaches are designed to be modular in that they are trained separately from the baseline model used to generate document embeddings. As an additional experiment, we use the CNN baseline to compare the performance of the modular setup to an end-to-end setup in which we integrate the RNN/self-attention/CRF layers directly onto the end of the CNN and train the both parts together.

All methods are trained using a batch size of 64 and the Adam optimizer [42] with learning rate of 1E-4. For each method, we train on the train set and then measure accuracy on the validation set after each epoch. We stop training when the validation accuracy fails to improve for five consecutive epochs. We save the model parameters after the epoch with the highest validation accuracy and use those to evaluate on our test set.

### Evaluation metrics

For each of our six classification tasks, we evaluate performance using two metrics—accuracy and macro F-score. We calculate macro F-score as follows:

$$\begin{array}{c} \begin{array}{cc} {\text{Precision}}_{c} & =\frac{\text{True}\;{\text{Pos}}_{c}}{\text{True}\;{\text{Pos}}_{c}+\text{False}\;{\text{Pos}}_{c}}\\ {\text{Recall}}_{c} & =\frac{\text{True}\;{\text{Pos}}_{c}}{\text{True}\;{\text{Pos}}_{c}+\text{False}\;{\text{Neg}}_{c}}\\ \text{F}1\;{\text{Score}}_{c} & =\frac{2\times {\text{Precision}}_{c}\times {\text{Recall}}_{c}}{{\text{Precision}}_{c}+{\text{Recall}}_{c}}\\ \text{Macro}\;\text{F}1\;\text{Score} & =\frac{1}{n}\sum_{c=i}^{n}\text{F}1\;{\text{Score}}_{c} \end{array} \end{array}$$ (8)

where *n* is the total number of possible classes within a given classification task and *c* is a specific class.

In any given task, accuracy measures the overall performance of each classifier across all possible classes, and it does not disproportionally penalize the classifier for underperforming on any one particular class. We note that in classification tasks such as ours in which each report is assigned to exactly one class, accuracy is the same as micro F-score.

On the other hand, macro F-score is heavily influenced by the performance on the minority classes. Therefore, macro F-score is an important metric because the distribution of label occurrences is highly skewed in many of our tasks—a more detailed breakdown of instances per label for each task is available in S1 Fig of our supporting information. When extracting information from clinical reports, it is generally important to accurately identify occurrences of rare medical conditions even if they do not appear very often. For both accuracy and F-score, we establish 95% confidence intervals using a data bootstrapping procedure [43] that is described in greater detail in our supporting information.

### Results

Our experimental results are displayed in Table 1 for the CNN baseline and in Table 2 for the HiSAN baseline. Across both the CNN and HiSAN baselines, all five methods of capturing case-level context achieve significantly better accuracy than the baseline of not utilizing any case-level context at all. In the unidirectional case where each classifier can only access context from previous reports, self-attention with linear-chain CRF achieves the overall best accuracy and macro F-scores. In the bidirectional case where each classifier can access both past and future reports, self-attention achieves the overall best accuracy while self-attention with linear-chain CRF achieves the best overall macro F-scores.

**Table 1:** Accuracy and macro F-Score (with 95% confidence intervals) of our different methods to capture case-level context on six different classification tasks using the CNN as the baseline. The top row is our baseline without any report level context, the middle group shows results of methods than can access both future and previous reports in a sequence, and the bottom group show results of methods that can only access previous reports in a sequence.

	Site	Subsite	Laterality	Histology	Behavior	Grade
**CNN—Baseline**	89.07	59.82	89.64	73.82	96.91	71.94
Accuracy	(88.91, 89.21)	(59.57, 60.05)	(89.49, 89.79)	(73.59, 74.03)	(96.82, 96.99)	(71.72, 72.15)
	56.22	24.33	46.91	22.79	67.16	73.72
Macro F-Score	(55.45, 56.83)	(23.92, 24.89)	(46.01, 47.83)	(22.42, 23.46)	(65.52, 68.93)	(72.24, 74.95)
**CNN w/ Concat All**	91.95	64.17	92.44	78.26	98.49	80.13
Accuracy	(91.74, 92.01)	(63.72, 64.19)	(92.28, 92.54)	(78.12, 78.54)	(98.40, 98.52)	(79.84, 80.22)
	58.62	22.57	**51.81**	21.67	74.58	75.21
Macro F-Score	(57.91, 59.14)	(22.08, 22.75)	**(51.19, 53.02)**	(21.23, 22.11)	(71.85, 77.44)	(74.74,79.72)
**CNN w/ Bi-RNN**	92.37	63.16	92.28	79.59	98.61	79.72
Accuracy	(91.87, 92.37)	(62.78, 63.68)	(92.01, 92.51)	(78.90, 79.63)	(98.48, 98.70)	(79.26, 80.00)
	62.14	27.42	49.89	32.29	73.83	79.03
Macro F-Score	(60.26, 62.66)	(26.16, 27.46)	(46.91, 50.33)	(30.65, 32.56)	(69.56, 78.16)	(78.66, 80.02)
**CNN w/ Bi-RNN + CRF**	92.26	63.03	92.29	79.27	98.64	80.66
Accuracy	(91.97, 92.45)	(62.65, 63.59)	(92.21, 92.69)	(78.62, 79.35)	(98.50, 98.71)	(80.53, 81.27)
	64.17	32.88	47.22	33.85	76.22	79.31
Macro F-Score	(63.89, 66.79)	(31.87, 33.49)	(44.75, 51.84)	(33.88, 35.78)	(74.28, 82.98)	(78.79, 80.20)
**CNN w/ Self-Attention**	**92.60**	**64.40**	**92.49**	**80.55**	98.73	**82.68**
Accuracy	**(92.32, 92.79)**	**(63.94, 64.84)**	**(92.22, 92.67)**	**(79.89, 80.66)**	(98.57, 98.78)	**(82.19, 82.87)**
	61.92	30.20	47.52	35.27	71.48	**82.55**
Macro F-Score	(60.75, 62.80)	(29.73, 31.20)	(46.36, 50.64)	(34.02, 35.73)	(70.06, 79.63)	**(81.59, 82.70)**
**CNN w/ Self-Att + CRF**	92.30	62.53	92.15	78.81	**98.79**	82.08
Accuracy	(92.12, 92.60)	(62.15, 63.06)	(91.95, 92.45)	(78.33, 79.07)	**(98.72, 98.92)**	(82.18, 82.87)
	**65.41**	**34.46**	49.29	**37.62**	**79.22**	81.27
Macro F-Score	**(64.67, 67.70)**	**(33.00, 34.62)**	(46.16, 53.53)	**(36.09, 37.81)**	**(73.90, 82.66)**	(80.79, 82.13)
**CNN w/ Concat Previous**	90.42	**62.20**	91.47	76.20	97.78	75.52
Accuracy	(90.34, 90.62)	**(61.94, 62.39)**	(91.29, 91.57)	(75.85, 76.26)	(97.73, 97.88)	(75.42, 75.84)
	56.53	22.25	47.43	20.41	67.44	77.62
Macro F-Score	(55.86, 57.11)	(21.90, 22.68)	(46.22, 48.10)	(20.15, 21.02)	(66.81, 70.65)	(73.61, 78.28)
**CNN w/ RNN**	90.60	61.88	91.43	76.01	97.96	76.49
Accuracy	(90.39, 90.91)	(60.99, 61.92)	(91.20, 91.73)	(75.55, 76.32)	(97.81, 98.07)	(76.15, 76.93)
	56.78	26.11	45.73	28.79	71.15	76.80
Macro F-Score	(55.68, 58.01)	(24.84, 26.10)	(44.30, 52.28)	(28.03, 29.77)	(69.74, 78.59)	(75.77, 77.22)
**CNN w/ RNN + CRF**	90.82	61.50	91.37	76.53	98.32	77.23
Accuracy	(90.56, 91.09)	(60.73, 61.63)	(91.25, 91.78)	(76.07, 76.85)	(98.18, 98.41)	(76.98, 77,72)
	60.19	30.24	47.65	32.57	73.05	76.11
Macro F-Score	(59.01, 61.86)	(29.71, 31.37)	(45.04, 48.61)	(31.21, 33.01)	(69.10, 78.35)	(75.92, 77.29)
**CNN w/ Masked Self-Att**	90.63	61.72	91.35	76.66	98.19	76.88
Accuracy	(90.36, 90.88)	(60.89, 61.82)	(90.90, 91.45)	(75.92, 76.71)	(97.91, 98.17)	(76.33, 77.13)
	59.48	29.42	47.44	30.67	71.33	76.69
Macro F-Score	(57.80, 60.40)	(27.78, 30.30)	(45.02, 49.31)	(29.53, 31.32)	(68.46, 77.09)	(75.68, 77.12)
**CNN w/ M. Self-Att + CRF**	**91.06**	62.00	**91.84**	**77.08**	**98.40**	**80.54**
Accuracy	**(90.88, 91.41)**	(61.55, 62.42)	**(91.38, 91.89)**	**(76.50, 77.27)**	**(98.32, 98.54)**	**(80.14, 80.88)**
	**61.09**	**30.98**	**48.14**	**33.95**	**78.66**	**79.92**
Macro F-Score	**(60.20, 63.52)**	**(30.71, 32.37)**	**(47.10, 51.60)**	**(32.86, 34.69)**	**(71.72, 80.88)**	**(79.09, 80.43)**

**Table 2:** Accuracy and macro F-Score (with 95% confidence intervals) of our different methods to capture case-level context on six different classification tasks using the HiSAN as the baseline. The top row is our baseline without any report level context, the middle group shows results of methods than can access both future and previous reports in a sequence, and the bottom group show results of methods that can only access previous reports in a sequence.

	Site	Subsite	Laterality	Histology	Behavior	Grade
**HiSAN—Baseline**	90.06	61.94	89.97	75.00	96.88	73.10
Accuracy	(89.90, 90.20)	(61.71, 62.17)	(89.81, 90.12)	(74.78, 75.21)	(96.80, 96.96)	(72.87, 73.30)
	62.98	30.31	51.46	33.20	79.73	74.45
Macro F-Score	(62.07, 63.69)	(29.95, 31.10)	(50.64, 52.37)	(32.36, 33.88)	77.23, 81.89)	(72.80, 75.79)
**HiSAN w/ Bi-RNN**	92.71	67.07	93.11	80.50	98.86	84.37
Accuracy	(92.49, 92.96)	(66.83, 67.69)	(92.78, 93.26)	(80.01, 80.75)	(98.85, 99.04)	(84.50, 85.17)
	67.63	37.26	52.72	38.26	82.81	83.69
Macro F-Score	(65.69, 68.57)	(35.88, 37.69)	(51.24, 56.81)	(37.74, 39.77)	(77.36, 86.03)	(83.29, 84.82)
**HiSAN w/ Bi-RNN + CRF**	92.44	66.66	92.59	79.82	98.75	84.35
Accuracy	(92.25, 92.75)	(66.10, 66.98)	(92.34, 92.80)	(79.61, 80.34)	(98.61, 98.82)	(83.79, 84.46)
	67.92	39.54	53.17	41.62	83.42	83.80
Macro F-Score	(66.61, 69.39)	(37.81, 39.81)	(51.40, 56.83)	(39.75, 41.74)	(80.42, 86.70)	(83.00, 84.20)
**HiSAN w/ Self-Attention**	**93.03**	**68.03**	**93.48**	**81.03**	**98.98**	**85.72**
Accuracy	**(92.99, 93.47)**	**(67.72, 68.61)**	**(93.15, 93.62)**	**(80.64, 81.37)**	**(98.88, 99.06)**	**(85.44, 86.07)**
	68.04	39.01	**55.56**	38.70	85.98	**86.12**
Macro F-Score	(65.91, 68.25)	(37.44, 39.23)	**(51.97, 61.50)**	(38.06, 39.98)	(82.13, 89.89)	**(85.76, 86.57)**
**HiSAN w/ Self-Att + CRF**	92.52	66.83	92.80	80.36	98.96	84.97
Accuracy	(92.34, 92.83)	(66.54, 67.44)	(92.59, 93.05)	(80.01, 80.74)	(98.79, 98.99)	(84.44, 85.09)
	**68.17**	**40.70**	54.74	**43.12**	**87.67**	85.35
Macro F-Score	**(66.77, 69.66)**	**(39.59, 41.47)**	(52.77, 57.99)	**(42.58, 44.56)**	**(81.70, 89.35)**	(84.56, 85.51)
**HiSAN w/ RNN**	91.37	64.13	91.81	77.08	98.24	79.15
Accuracy	(91.18, 91.70)	(64.06, 64.96)	(91.71, 92.21)	(76.56, 77.30)	(98.14, 98.38)	(78.77, 79.49)
	63.59	34.50	46.81	33.42	79.54	79.22
Macro F-Score	(62.40, 65.41)	(32.61, 34.83)	(47.53, 51.81)	(33.19, 35.18)	(74.15, 82.77)	(78.60, 79.96)
**HiSAN w/ RNN + CRF**	91.92	65.56	92.38	77.76	98.61	81.80
Accuracy	(91.53, 92.03)	(65.14, 65.99)	(92.29, 92.78)	(77.43, 78.18)	(98.43, 98.66)	(81.82, 82.55)
	65.62	36.99	50.38	38.76	85.25	81.58
Macro F-Score	(64.84, 67.95)	(36.45, 38.43)	(49.79, 59.41)	(38.43, 40.45)	(77.71, 86.18)	(81.10, 82.32)
**HiSAN w/ Masked Self-Att**	91.50	64.82	91.94	77.54	98.20	79.38
Accuracy	(91.26, 91.77)	(64.56, 65.42)	(91.87, 92.37)	(77.13, 77.86)	(98.15, 98.39)	(79.12, 79.86)
	63.81	35.32	50.34	36.00	81.77	80.29
Macro F-Score	(62.66, 65.73)	(34.07, 35.88)	(49.09, 55.02)	(34.91, 36.93)	(76.55, 84.38)	(79.27, 80.55)
**HiSAN w/ M. Self-Att + CRF**	**92.11**	**65.57**	**92.66**	**79.22**	**98.85**	**83.64**
Accuracy	**(91.75, 92.24)**	**(65.47, 66.32)**	**(92.45, 92.92)**	**(78.74, 79.49)**	**(98.65, 98.88)**	**(83.10, 83.77)**
	**65.69**	**37.85**	**52.22**	**39.17**	**86.10**	**83.00**
Macro F-Score	**(64.82, 67.96)**	**(37.21, 39.17)**	**(50.93, 54.85)**	**(37.16, 39.28)**	**(81.15, 88.75)**	**(81.53, 83.86)**

To further confirm the statistical significance of utilizing case-level context, we utilized McNemar’s test [44], which generates a p-value indicating if two machine learning classifiers have a different proportion of errors on the test set. We compared the predictions of each method of capturing case-level context against the baseline model predictions without case-level context; we compared each method using both the CNN and HiSAN, with and without future reports, and on each of the six tasks. In all 108 comparisons between the method for capturing case-level context and the baseline, McNemar’s test generated a p-value of <0.0001, indicating with strong statistical significance that case-level context makes a difference in test set accuracy.

Across all methods, the unidirectional approach in which the network can only access earlier reports performs worse than the bidirectional approach in which the network can access both earlier and future reports. This makes intuitive sense because the unidirectional approaches have access to less information. In our particular application, the ground truth labels are identified based off all reports in the sequence; therefore, for any given report, future reports may be relevant for accurately predicting the ground truth label. Despite this, our results show that the unidirectional approaches still significantly outperform the baseline of no case-level context.

Once again, we utilize McNemar’s test to confirm the statistical significance of the difference between unidirectional and bidirectional approaches. For each method, we compare the unidirectional results against the results of the bidirectional counterpart; this is done for both the CNN and HiSAN on each of the six tasks. Out of 54 comparisons, McNemar’s test generated a p-value of <0.0001 in all but five tests (see S1 Table of our supporting information for detailed results), indicating with strong statistical significance that the bidirectional approach gives different predictions on the test set than the unidirectional approach.

Our results in Tables 1 and 2 indicate that incorporating case-level context results in higher macro F-scores across all tasks than the baseline, indicating that case-level context improves performance on the rare classes. To further verify this, in S2 Table of our supporting information, we break down the performance by individual class label for the CNN and HiSAN without case-level context, with unidirectional case-level context (masked self-attention with CRF), and with bidirectional case-level context (self-attention with CRF) on site, laterality, histology, behavior, grade. We observe the general trend that across all tasks and the vast majority of classes, bidirectional case-level context gives the best f-score, unidirectional case-level context gives the second best, and no case-level context performs the worst—the few exceptions only occur in classes with extremely few training instances (mostly in classes that make up <0.2% of the training data).

In S3 Table of our supporting information, we also show the performance comparison of our modular methods with their end-to-end equivalents using the CNN baseline model. To attain the best performance in end-to-end training, we first pretrain the CNN portion of the model independently, then train the entire model (both the CNN and RNN/self-attention/CRF portions) using end-to-end training. Therefore, the main difference between the modular training method and the end-to-end training method is that in the end-to-end method, the CNN weights can be further fine-tuned during the contextual training portion.

Compared to modular two-step training, end-to-end training is neither consistently better nor worse in terms of accuracy and macro F-score; performance varies by task. Across the different tasks and approaches, modular training usually achieves within 1% relative accuracy compared to end-to-end training. We believe that these results support the view that users can utilize our modular approaches for capturing case-level context and attain similar or better performance compared to a more complicated end-to-end approach with an equivalent architecture.

## Discussion

As discussed in our methods section, deep learning approaches for text classification generally encode an input document into a document embedding representation, which is then used for classification purposes. Our methods to capture case-level context transform these document embeddings such that they account for information from other relevant reports in the sequence. We can visualize the document embeddings before and after our modular add-ons to better understand the transformations that are taking place.

**Fig 2:**
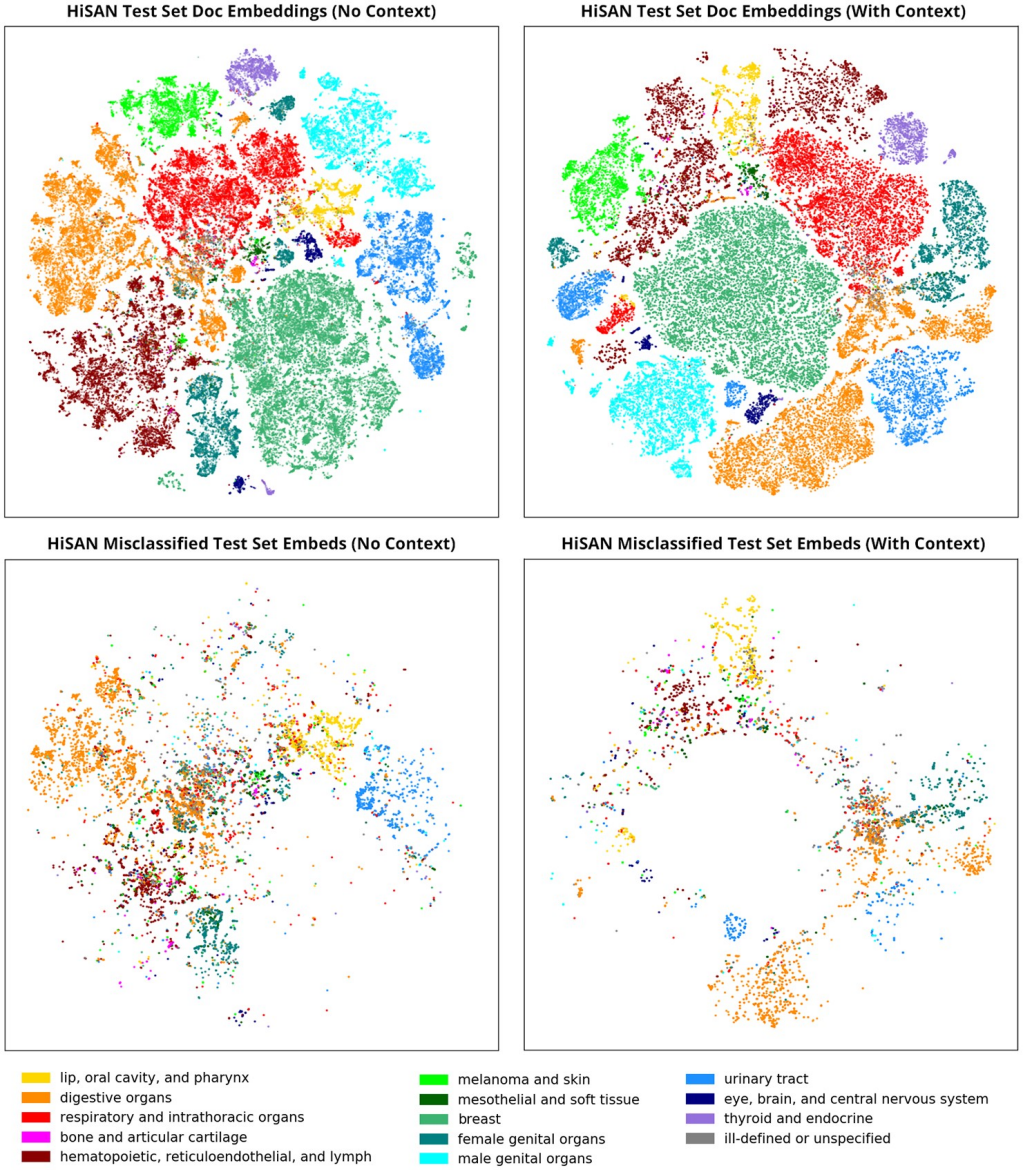
The top two subfigures show the cancer site document embeddings generated by the HiSAN for each pathology report in our test set with and without the self-attention module for capturing case-level context. The bottom two figures only show the document embeddings of misclassified reports in our test set. All document embeddings are colored by the ground truth organ system and visualized using t-SNE.

In Fig 2, we show the document embeddings of our pathology reports on the site task generated by the HiSAN without case-level context (left) and the HiSAN with the self-attention method for capturing case-level context (right). The top pair of subfigures visualizes all document embeddings from our test set, colored by the ground truth organ system. We notice that clusters generated by the HiSAN with case-level context are slightly cleaner than the baseline HiSAN—there is less overlap between clusters and there are fewer subclusters within each organ system. This suggests that adding case-level context improves the HiSAN’s ability to distinguish between pathology reports belonging to different organ systems.

The bottom pair of subfigures show only the document embeddings of misclassified reports in the test set, colored by the ground truth organ system. This visualization allows us to better understand the types of errors that each approach makes. Based off the figure, we observe two general types of errors: (1) within-cluster misclassifications, in which the misclassified report is still clustered in the correct organ system, and (2) out-of-cluster misclassifications, in which the misclassified report is placed in an incorrect organ system. We see that adding document-level context reduces out-of-cluster errors compared to the baseline.

To gain a more in-depth understanding of the nature of the errors in our experiments, we randomly sampled 200 pathology reports that were misclassified by the baseline HiSAN (no case-level context) and manually examined the text of the pathology report. We then added the self-attention modular add-on and reclassified the same 200 reports to see which types of errors are resolved by incorporating case-level context.

Based off our manual examination, we identified two general categories of errors, which respectively correspond with the out-of-cluster and in-cluster misclassifications in Fig 2. In the first category of errors, the report either (1) does not appear to contain any information associated with the ground truth site or (2) mentions two or more (usually metastatic) sites; this is most likely because the report is an addendum or biopsy of a secondary or metastatic site. The baseline HiSAN therefore mispredicts the (non-ground truth) site that is mentioned in the report. Out of 200 randomly sampled reports, 80 reports fell into this category.

Adding case-level context can effectively deal with this type of error because the ground-truth label is almost always contained in another report in the sequence. Of the 80 reports misclassified by the baseline HiSAN in this first category, adding case-level context rectified 61 of the reports (76%).

In the second category of errors, the predicted site is a neighboring organ of the ground truth site or is within the same organ system as the ground truth site. Our manual analysis revealed that there is often overlap in the language used to describe organs within certain organ systems—for example, the ground truth site may be the rectosigmoid junction but the report may also mention the colon, or the ground truth site may be the cervix but the report may also mention the uterus. For these reports, we attempted to manually classify the site ourselves without knowing the ground truth site or the HiSAN’s predicted site, and more often than not we made the same prediction as the HiSAN; this indicates that language used in the reports is confusing not just for the HiSAN but also for an inexperienced human annotator. Four commonly confused groups of sites were (1) between C42 hematopoietic and reticuloendothelial systems, C44 skin, and C77 lymph nodes, (2) between C51 vulva, C52 vagina, C53 cervix, and C54 uterus, (3) between C64 kidney, C65 renal pelvis, C66 ureter, and C67 bladder, and (4) between C18 colon, C19 rectosigmoid junction, C20 rectum, and C21 anus.

This second category of errors also includes reports associated with ill-defined sites (C76), unknown sites (C80), or a general catch-all site for a particular organ system (e.g., C57 unspecified female genital organs). In these reports, the ground truth site is one of these ill-defined sites despite the report mentioning specific organs or cancer sites. Out of 200 misclassified reports examined, 120 reports fell into this second category.

Adding case-level context is less effective for dealing with this second category of errors because these confounding effects typically exist across all reports in the sequence; however, incorporating contextual clues from other reports may help narrow down the correct site. Of the 120 reports misclassified by the baseline HiSAN in this second category, adding case-level context rectified 35 of the reports (29%).

**Fig 3:**
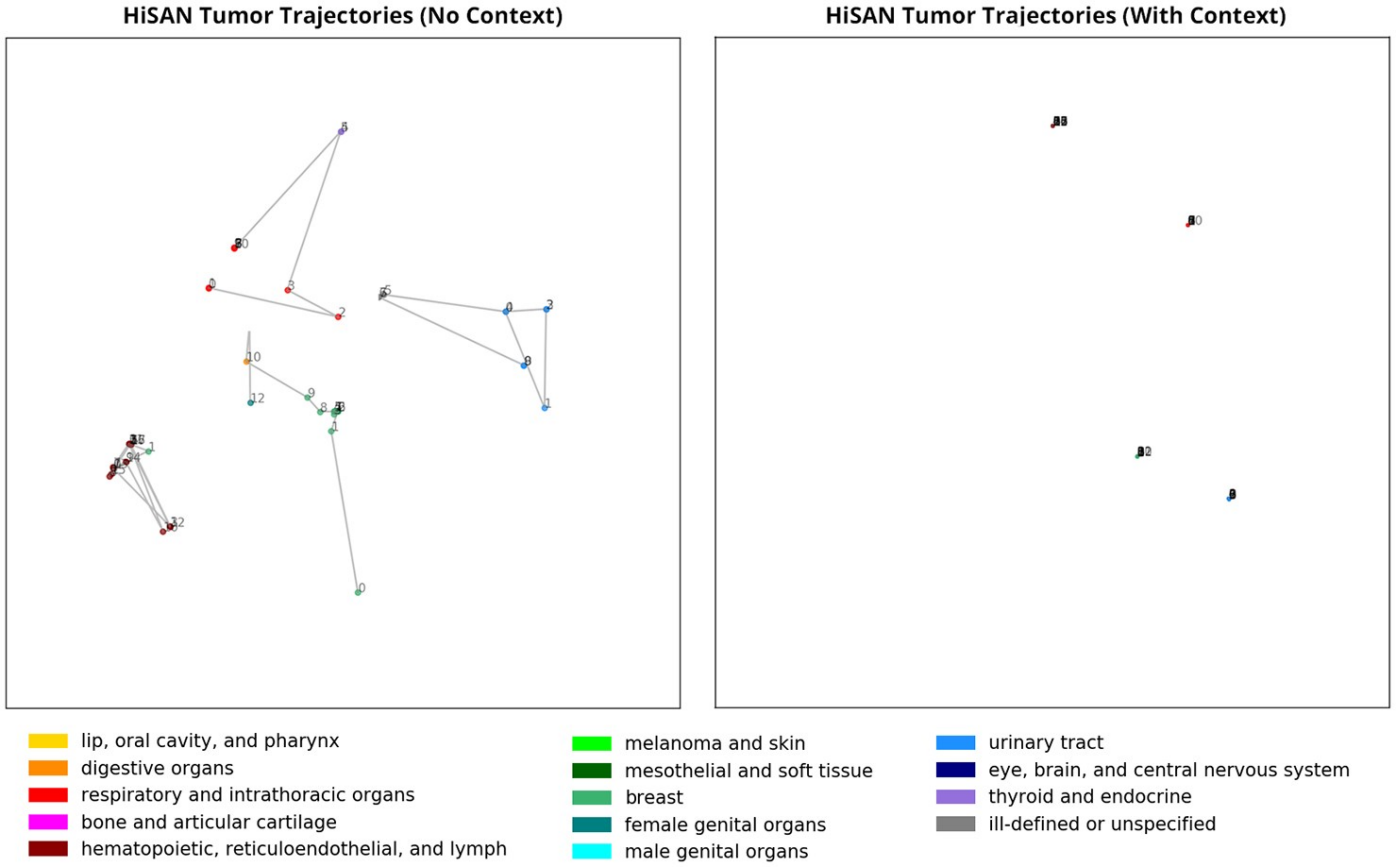
The cancer site document embeddings generated by the HiSAN for the pathology reports associated with four unique tumor IDs, with and without the self-attention module for capturing case-level context. These figures share the same axes as Fig 2 and thus can be directly compared. Within each of the four trajectories, document embeddings are numbered from earliest to latest and are colored by the predicted organ system. We notice that without case-level context, reports belonging to the same tumor ID are classified under different organ systems. Adding case-level context addresses this problem and all document embeddings from the same tumor ID are placed in the same location in the embedding space.

By visualizing the document embeddings from only the reports associated with a single tumor ID, we can show how adding case-level context affects the information captured in individual document embeddings. In Fig 3, we visualize the trajectories of the document embeddings belonging to four unique tumor IDs, colored by the *predicted* organ system. We see that the document embeddings generated by the HiSAN without case-level context are spread out over the embedding space—this is generally because each pathology report in a sequence may contain slightly different information, and as mentioned previously, multiple sites may be tested to check the spread of cancer to additional sites. Furthermore, there may be multiple different primary sites identified within the same tumor ID trajectory, likely because certain reports may contain information about secondary or metastatic sites. This is problematic because we wish to assign the same tumor-level labels to all reports belonging to the same tumor ID.

Once case-level context is incorporated, all document embeddings from the same tumor ID are placed in the exact same location—this is appropriate for our application because all pathology reports associated with the same tumor ID should have the exact same label. Furthermore, in the examples shown, all reports in the same trajectory are assigned the same label and thus misclassifications caused by secondary or metastatic sites are eliminated. We note that this type of trajectory analysis may be useful for identifying addendum-type and metastatic-type reports, which tend to be the pathology reports whose document embedding position shifts significantly and/or label changes once case-level context is included.

## Conclusion

In this paper, we showed how adding a modular component for capturing case-level context on top of an existing deep learning text classification model designed for individual documents can improve classification accuracy of aggregate-level labels for cancer pathology reports. We compared the performance of five methods for capturing case-level context—concatenation, RNNs, RNNs with linear-chain CRF, self-attention, and self-attention with linear-chain CRF—and showed that all five achieved better accuracy than the baseline of no case-level context across six classification tasks. In the unidirectional case where each classifier can only access context from previous reports, self-attention with linear-chain CRF achieves the overall best accuracy and macro F-scores. In the bidirectional case where each classifier can access both past and future reports, self-attention achieves the overall best accuracy while self-attention with linear-chain CRF achieves the best overall macro F-scores.

Other than concatenation, our approaches are designed as modular add-ons that are easy to train on top of an existing deep learning text classification model built for individual documents. We show that our modular design, which uses a two-step training approach, has very similar performance to an identical end-to-end architecture, which requires far more engineering and may be prohibitively expensive in terms of time and memory for complex baseline models.

In our experiments, we demonstrated the effectiveness of our approach in the application for cancer pathology reports, where a sequence of reports belonging to a unique tumor ID were all tagged with the same aggregate-level labels. We expect that with minimal modifications, our approaches can be applied towards a wide range of other EHR- and clinical text-based tasks. In future work, we plan to extend our experiments to clinical applications where each clinical report in a sequence is tagged with a different label, such as using a patient’s previous clinical notes to inform the extraction of diagnosis or treatment codes from a given clinical report. The code used for our experiments is available online at https://github.com/iamshang1/Projects/tree/master/Papers/Case_Level_Context.

### Detailed experimental procedures

#### Pathology report preprocessing procedure

1. Remove identifier segments (registry ID, patient ID, tumor number, and document ID)
2. Remove XML tags
3. Lowercase
4. Replace tabs with spaces, but retain line breaks
5. Remove periods in the abbreviations “dr.”, “am.”, and “pm.” (this allows splitting lines by periods later)
6. Remove periods in floats by replacing all instances of floats with the string “floattoken” (this allows splitting lines by periods later)
7. Replace all integers higher than 100 with the string “largeinttoken” (to reduce the number of unique tokens associated with numbers)
8. Convert unicode to ASCII
9. If the same non-alphanumeric character appears consecutively more than once, replace it with a single copy of that character
10. Add a space before and after every non-alphanumeric character
11. Replace any token that appears less than 5 times across the entire corpus with the string “unknowntoken”
12. For the HiSAN input, split the document by naturally occurring linebreaks.
13. For the HiSAN input, split lines longer than 50 words by any character in the Linebreak Characters Set 1 (listed below)
14. For the HiSAN input, split lines still longer than 50 words by any character in the Linebreak Characters Set 2 (listed below)
15. Replace each word token with the appropriate Word2Vec embedding

#### Linebreak characters set 1

- .
- :
- ;
- /
- ?
- ~
*
- <
- #

#### Linebreak characters set 2

- any standalone single letter except ‘a’ (many reports use single letters to itemize lists)
- ,
-
- _
- =

#### Bootstrapping procedure for confidence interval

1 For each model and classification task, save the model’s predictions on the test set (hereon referred to as the original predictions)
2 Randomly select predicted labels (with replacement) from the original predictions to create a new set of predicted labels of the same size as the test set (hereon referred to as bootstrapped set)
3 Calculate accuracy and macro F-score on bootstrapped set
4 Repeat steps (2) and (3) 1000 times, saving the scores each time
5 Calculate the 95% confidence interval for accuracy and macro F-score by finding the 2.5 and 97.5 percentile entry for that metric within the 1000 runs (since F-score is not normally distributed)

## Supporting information

S1 Fig (a) Histograms of the number of occurrences per label for each of the six classification tasks, arranged from most common to least common. For the site, subsite, and histology tasks, we only show the 50 most common labels. Detailed information about each label can be found online in the SEER coding manual at https://seer.cancer.gov/tools/codingmanuals/. (b) Histograms of the number of occurrences per label for each of the six classification tasks, arranged from most common to least common. For the site, subsite, and histology tasks, we only show the 50 most common labels. Detailed information about each label can be found online in the SEER coding manual at https://seer.cancer.gov/tools/codingmanuals/.
(TIF)

S2 Fig Histogram of number of pathology reports associated with each unique tumor ID. (TIF)

S1 Table McNemar’s tests of statistical significance. (PDF)

S2 Table Case-level context F-score breakdown by class. (PDF)

S3 Table Modular vs end-to-end training. (PDF)
