# Supplementary figures and images for: Using case-level context to classify cancer pathology reports
Source: PLoS One. 2020 May 12;15(5):e0232840. doi: 10.1371/journal.pone.0232840 (PMC7217446; doi:10.1371/journal.pone.0232840)

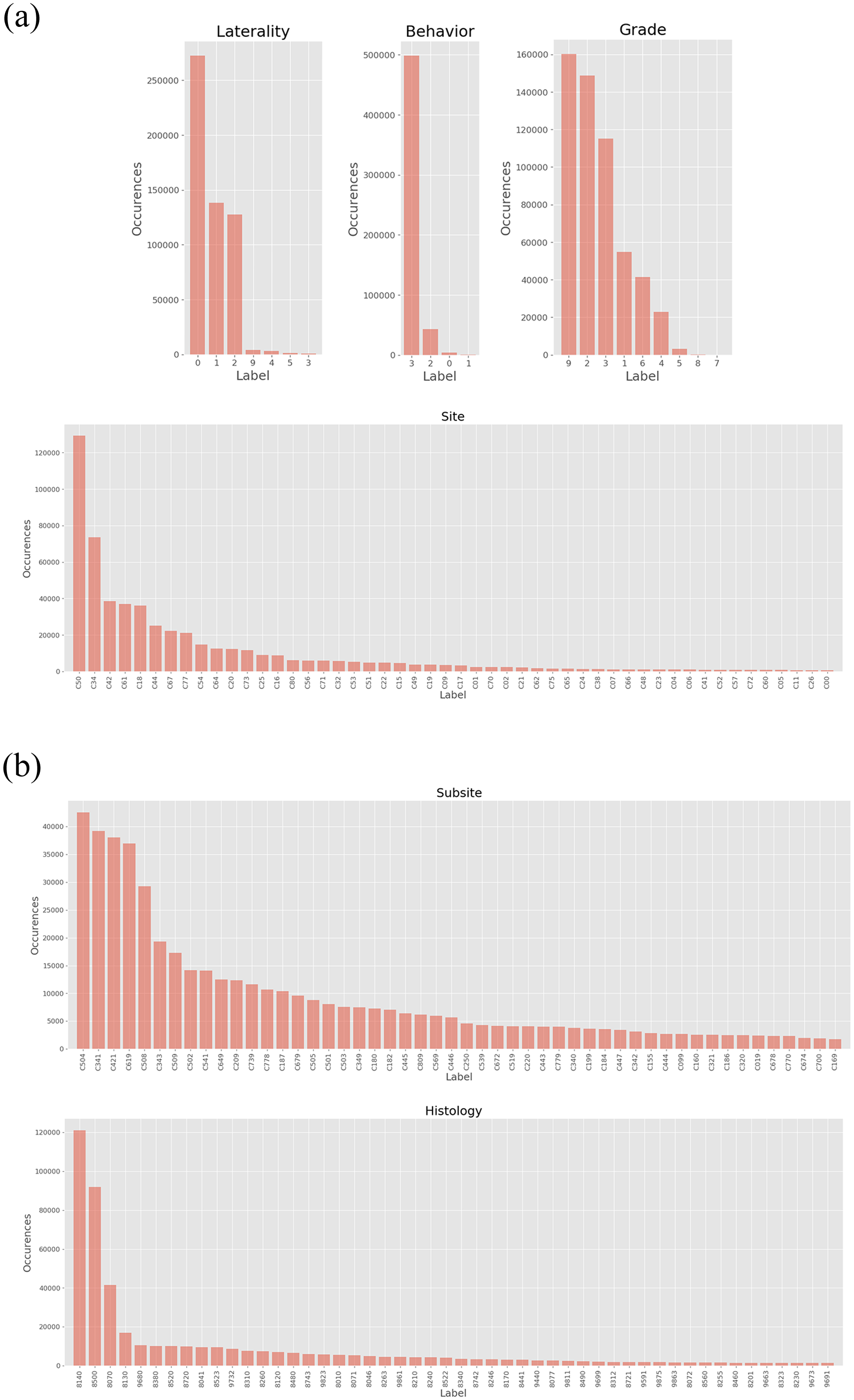

Supplement: S1 Fig — (a) Histograms of the number of occurrences per label for each of the six classification tasks, arranged from most common to least common. For the site, subsite, and histology tasks, we only show the 50 most common labels. Detailed information about each label can be found online in the SEER coding manual at https://seer.cancer.gov/tools/codingmanuals/. (b) Histograms of the number of occurrences per label for each of the six classification tasks, arranged from most common to least common. For the site, subsite, and histology tasks, we only show the 50 most common labels. Detailed information about each label can be found online in the SEER coding manual at https://seer.cancer.gov/tools/codingmanuals/. (TIF) [file pone.0232840.s001.tif]

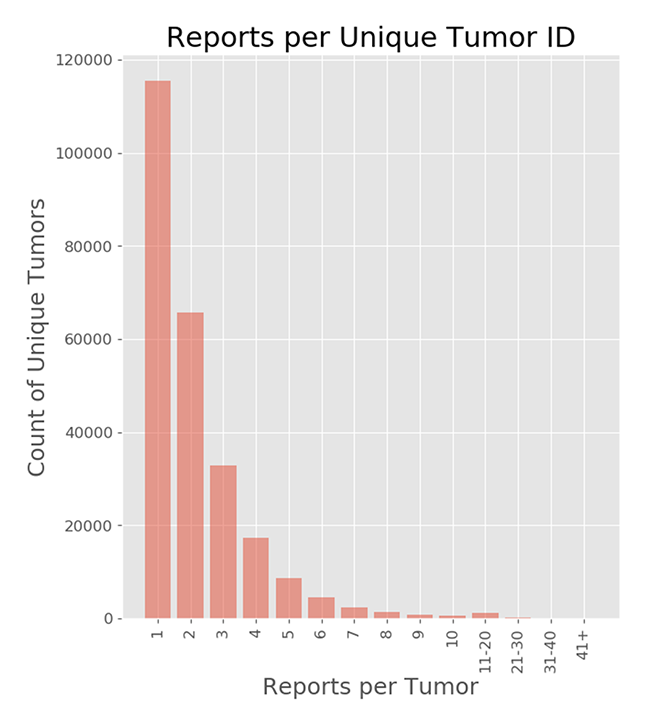

Supplement: S2 Fig — (TIF) [file pone.0232840.s002.tif]
