# Supplementary material for: Using case-level context to classify cancer pathology reports
Source: PLoS One. 2020 May 12;15(5):e0232840. doi: 10.1371/journal.pone.0232840 (PMC7217446; doi:10.1371/journal.pone.0232840)
Supplement: S1 Table — (PDF) [file pone.0232840.s003.pdf]

McNemar's tests of statistical significance

Table S1. For each task and each method for capturing case-level context, we use McNemar's test to generate p-values that show the statistical significance of the difference in performance between the unidirectional (previous reports only) vs bidirectional (previous and future reports) approach.

	**Site**	**Subsite**	**Laterality**	**Histology**	**Behavior**	**Grade**
**CNN Concat Prev vs CNN Concat All**	<0.0001	<0.0001	<0.0001	<0.0001	<0.0001	<0.0001
**CNN w/ RNN vs CNN w/ Bi-RNN**	<0.0001	<0.0001	<0.0001	<0.0001	<0.0001	<0.0001
**CNN w/ RNN + CRF vs CNN w/ Bi-RNN + CRF**	<0.0001	<0.0001	<0.0001	<0.0001	<0.0001	<0.0001
**CNN w/ Masked SA vs CNN w/ SA**	<0.0001	<0.0001	<0.0001	<0.0001	<0.0001	<0.0001
**CNN w/ Masked SA + CRF vs CNN w/ SA + CRF**	<0.0001	<0.0001	**0.0084**	<0.0001	<0.0001	<0.0001
**HiSAN w/ RNN vs HiSAN w/ Bi-RNN**	<0.0001	<0.0001	<0.0001	<0.0001	<0.0001	<0.0001
**HiSAN w/ RNN + CRF vs HiSAN w/ Bi-RNN + CRF**	<0.0001	<0.0001	**0.1073**	<0.0001	**0.1047**	<0.0001
**HiSAN w/ Masked SA vs HiSAN w/ SA**	<0.0001	<0.0001	<0.0001	<0.0001	<0.0001	<0.0001
**HiSAN w/ Masked SA + CRF vs HiSAN w/ SA + CRF**	<0.0001	<0.0001	**0.2273**	<0.0001	**0.3182**	<0.0001

Note: For each task and each method for capturing case-level context, we also used McNemar's test to generate p-values that show the statistical significance of the difference in performance between the method for capturing case-level context and the baseline of no case-level context. We do not include a table for these results because for every McNemar's test, the p-value was <0.0001.
