# Supplementary material for: Using case-level context to classify cancer pathology reports
Source: PLoS One. 2020 May 12;15(5):e0232840. doi: 10.1371/journal.pone.0232840 (PMC7217446; doi:10.1371/journal.pone.0232840)
Supplement: S2 Table — (PDF) [file pone.0232840.s004.pdf]

Case-level context F-score breakdown by class

Table S2. CNN and HiSAN f-scores for each task by class with and without report-level context. F-scores are calculated using a one-vs-all setup for the class being examined. For histology, we only show the 20 most common classes in the test set.

Site	% Total Train Set	% Total Test Set	CNN	CNN + M. SA-CRF	CNN SA-CRF	HiSAN	HiSAN + M. SA-CRF	HiSAN + SA-CRF
C00	0.10	0.07	81.20	84.06	**89.92**	84.29	89.05	**96.35**
C01	0.47	0.41	53.90	61.56	**67.81**	53.23	66.98	**71.98**
C02	0.47	0.42	78.38	82.17	**90.24**	80.26	85.49	**89.96**
C03	0.07	0.10	39.51	66.67	**71.55**	68.85	**84.47**	81.70
C04	0.19	0.19	66.18	71.27	**79.12**	75.65	83.24	**83.94**
C05	0.15	0.10	61.19	67.00	**72.20**	68.10	78.15	**82.79**
C06	0.20	0.13	58.66	**73.93**	73.12	68.33	**73.03**	72.48
C07	0.19	0.17	68.88	77.96	**81.18**	77.21	83.20	**87.17**
C08	0.05	0.07	17.65	36.21	**38.98**	37.50	33.58	**43.10**
C09	0.67	0.58	60.27	67.99	**76.17**	63.30	73.95	**77.38**
C10	0.11	0.13	11.34	**21.37**	16.96	20.79	**24.47**	21.46
C11	0.14	0.10	47.56	50.26	**52.36**	58.89	55.67	**63.00**
C12	0.07	0.04	46.15	33.85	**58.82**	36.62	49.38	**71.11**
C13	0.10	0.05	24.10	18.90	**27.27**	33.33	40.71	**53.61**
C14	0.08	0.13	7.36	**18.90**	12.21	10.17	11.58	**14.61**
C15	0.74	0.89	70.31	75.42	**79.67**	76.99	**79.05**	79.02
C16	1.74	1.54	75.10	80.14	**84.15**	79.20	82.54	**84.50**
C17	0.60	0.50	64.48	72.60	**74.75**	72.18	76.36	**79.44**
C18	6.61	6.31	89.18	92.88	**93.32**	90.03	94.41	**94.60**
C19	0.66	0.66	38.22	47.62	**48.64**	39.15	52.05	**53.55**
C20	2.09	2.36	79.79	84.94	**87.02**	81.19	85.95	**86.89**
C21	0.32	0.35	74.14	80.00	**80.33**	80.16	81.15	**84.85**
C22	0.78	0.60	68.84	72.44	**77.12**	71.96	74.90	**77.43**
C23	0.15	0.20	66.11	73.33	**75.32**	66.30	**77.42**	74.69
C24	0.21	0.21	38.73	45.18	**55.22**	45.69	59.54	**61.43**
C25	1.26	1.28	81.11	85.75	**88.39**	83.64	88.01	**89.52**
C26	0.10	0.20	2.65	**11.50**	10.79	7.51	12.88	**14.02**
C30	0.12	0.05	56.06	54.55	**57.35**	54.14	61.54	**70.69**
C31	0.09	0.05	28.21	**48.10**	42.22	40.40	46.30	**52.43**
C32	0.95	0.84	84.39	88.58	**93.14**	87.18	89.82	**92.62**
C33	0.02	0.02	8.70	18.18	**33.33**	26.67	25.81	**33.33**
C34	13.74	12.87	91.84	94.88	**96.14**	92.98	95.71	**96.19**
C37	0.04	0.04	18.18	51.35	**51.95**	**50.00**	43.84	44.16
C38	0.23	0.23	58.22	67.38	**80.35**	60.85	66.09	**72.54**
C40	0.10	0.08	53.73	48.00	**53.97**	64.63	72.48	**75.95**
C41	0.17	0.16	6.64	16.59	**17.52**	29.71	**35.11**	31.40
C42	7.48	8.97	93.01	95.19	**95.29**	93.97	94.87	**95.11**
C44	4.36	4.31	94.54	95.79	**96.19**	95.03	96.66	**97.04**
C47	0.04	0.02	24.24	21.82	**39.34**	26.67	26.67	**30.77**
C48	0.21	0.24	12.58	21.34	**29.59**	24.46	36.45	**53.08**
C49	0.70	0.68	63.40	67.63	**70.54**	69.06	73.65	**73.95**
C50	27.99	27.66	98.72	99.37	**99.48**	98.89	99.37	**99.52**
C51	0.82	0.71	90.84	94.33	**95.53**	93.66	95.73	**96.05**
C52	0.12	0.11	46.59	66.67	**74.04**	63.77	72.12	**75.93**
C53	1.13	0.63	81.04	89.45	**91.18**	85.63	90.80	**91.50**
C54	2.74	2.64	91.31	94.25	**96.11**	92.72	95.57	**95.71**
C55	0.07	0.07	0.00	0.00	0.00	10.26	**21.51**	15.76
C56	1.10	1.08	73.46	77.33	**80.05**	75.49	81.23	**84.40**
C57	0.13	0.21	10.33	14.10	**25.85**	26.90	40.22	**45.25**
C60	0.13	0.13	94.81	96.86	**98.62**	91.41	97.53	**99.31**
C61	4.52	5.26	97.67	98.43	**98.75**	97.86	98.60	**98.71**
C62	0.23	0.17	82.90	86.21	**92.88**	89.08	93.56	**97.05**
C63	0.02	0.02	0.00	**16.00**	0.00	52.94	40.00	**76.47**
C64	1.40	1.18	89.27	91.43	**92.87**	90.43	93.33	**93.52**
C65	0.29	0.36	53.47	68.76	**68.97**	56.68	75.81	**79.50**
C66	0.19	0.28	40.49	**66.42**	66.10	54.44	**72.41**	68.82
C67	3.47	4.85	93.36	96.59	**96.70**	94.10	96.69	**96.91**
C68	0.11	0.13	9.27	**27.68**	25.00	26.37	28.57	**31.28**
C69	0.10	0.06	37.21	**65.04**	64.46	60.66	**75.20**	67.63

Table S2. (Continued) CNN and HiSAN f-scores for each task by class with and without report-level context. F-scores are calculated using a one-vs-all setup for the class being examined. For histology, we only show the 20 most common classes in the test set.

C70	0.22	0.15	87.27	87.54	**89.61**	88.00	90.97	**91.08**
C71	1.08	0.82	91.32	95.62	**96.57**	92.62	95.44	**96.33**
C72	0.08	0.04	44.07	43.68	**53.73**	45.57	56.00	**58.82**
C73	1.74	1.74	95.14	96.88	**98.06**	95.86	97.75	**98.59**
C74	0.09	0.07	52.10	56.20	**60.98**	59.68	64.86	**76.03**
C75	0.19	0.11	85.97	**88.70**	87.29	91.45	94.92	**96.67**
C76	0.05	0.03	0.00	0.00	0.00	0.00	0.00	0.00
C77	4.32	4.11	79.03	81.96	**83.52**	81.00	83.42	**83.67**
C80	1.11	1.03	20.64	22.36	**30.65**	24.29	31.73	**32.63**

**Lat.**	**% Total Train Set**	**% Total Test Set**	**CNN**	**CNN + M. SA-CRF**	**CNN SA-CRF**	**HiSAN**	**HiSAN + M. SA-CRF**	**HiSAN + SA-CRF**
0	46.76	48.46	93.95	94.99	**95.18**	94.30	95.55	**95.75**
1	26.81	26.12	86.60	90.29	**90.48**	87.07	91.38	**91.52**
2	24.81	24.02	86.17	89.71	**90.66**	86.87	91.62	**91.82**
3	0.11	0.07	0.00	0.00	0.00	0.00	0.00	0.00
4	0.60	0.53	16.99	26.25	**40.79**	36.76	42.38	**45.53**
5	0.15	0.22	33.64	48.74	**49.87**	45.75	**53.10**	51.95
9	0.76	0.57	2.68	**13.31**	10.72	5.16	**16.11**	12.94

**Beh.**	**% Total Train Set**	**% Total Test Set**	**CNN**	**CNN + M. SA-CRF**	**CNN SA-CRF**	**HiSAN**	**HiSAN + M. SA-CRF**	**HiSAN + SA-CRF**
0	0.38	0.22	82.49	84.19	**87.53**	84.27	83.66	**87.22**
1	0.09	0.08	6.74	**52.98**	35.71	56.55	**72.00**	63.83
2	7.58	7.83	81.08	90.12	**90.46**	81.56	91.74	**91.79**
3	91.95	91.88	98.31	99.11	**99.14**	98.28	99.25	**99.26**

**Hist.**	**% Total Train Set**	**% Total Test Set**	**CNN**	**CNN + M. SA-CRF**	**CNN SA-CRF**	**HiSAN**	**HiSAN + M. SA-CRF**	**HiSAN + SA-CRF**
8140	20.29	20.78	84.56	88.41	**89.73**	85.76	89.84	**90.57**
8500	19.68	20.22	88.22	90.81	**91.37**	88.82	91.61	**91.96**
8070	7.37	6.51	80.84	86.05	**86.96**	80.98	87.27	**88.26**
8130	2.61	3.77	83.55	84.24	**86.20**	83.90	87.54	**89.05**
8520	2.13	2.23	73.56	83.96	**85.47**	76.06	84.43	**85.24**
9680	2.15	2.20	76.07	81.67	**85.13**	78.07	85.67	**86.65**
9732	1.63	2.11	93.34	95.95	**96.44**	93.94	96.03	**96.60**
8523	2.12	1.81	41.18	48.83	**52.12**	46.51	53.33	**55.34**
8380	1.84	1.77	80.64	85.37	**88.08**	83.37	86.19	**88.95**
8720	1.61	1.74	71.31	76.47	**80.61**	70.54	82.65	**83.12**
8120	1.22	1.52	52.37	**66.11**	62.16	56.51	**66.81**	66.69
8041	1.51	1.32	82.09	88.08	**89.22**	79.22	89.76	**91.22**
8480	1.30	1.29	56.38	63.06	**66.15**	57.81	67.92	**69.57**
9861	0.91	1.26	70.45	72.44	**73.73**	72.75	73.30	**74.00**
9823	1.05	1.19	83.59	85.56	**88.73**	84.31	89.00	**89.15**
8743	1.02	1.14	71.37	84.30	**86.67**	71.41	87.22	**87.58**
8071	0.99	1.07	60.61	**68.98**	68.61	57.20	71.77	**73.74**
8260	1.05	1.05	71.66	74.97	**79.31**	71.92	**78.86**	78.47
8522	0.95	0.89	39.10	45.17	**49.36**	40.90	52.82	**57.85**
9811	0.46	0.85	79.27	91.04	**93.89**	85.48	94.00	**94.39**

**Grade**	**% Total Train Set**	**% Total Test Set**	**CNN**	**CNN + M. SA-CRF**	**CNN SA-CRF**	**HiSAN**	**HiSAN + M. SA-CRF**	**HiSAN + SA-CRF**
1	9.71	10.46	64.01	73.21	**73.47**	64.17	76.18	**76.75**
2	27.29	28.32	71.45	80.41	**80.89**	72.55	83.00	**83.84**
3	22.40	19.90	67.47	77.25	**77.65**	68.74	80.23	**80.87**
4	3.88	4.94	54.27	**60.48**	64.21	60.02	65.51	**65.73**
5	0.70	0.64	80.44	86.38	**90.62**	82.25	90.56	**90.77**
6	8.14	9.31	89.75	92.16	**93.03**	90.89	93.05	**93.22**
8	0.04	0.02	80.00	93.33	**95.65**	88.37	**93.62**	88.37
9	27.84	26.41	72.30	78.11	**78.25**	73.54	80.08	**81.07**
