# Supplementary material for: Using case-level context to classify cancer pathology reports
Source: PLoS One. 2020 May 12;15(5):e0232840. doi: 10.1371/journal.pone.0232840 (PMC7217446; doi:10.1371/journal.pone.0232840)
Supplement: S3 Table — (PDF) [file pone.0232840.s005.pdf]

Modular vs end-to-end training

Table S3. Accuracy and macro F-Score (with 95% confidence intervals) of modular and end-to-end training using the CNN baseline model. Results are grouped in pairs – for each technique for capturing report level context, we compare the modular results with the end-to-end results.

	Site	Subsite	Laterality	Histology	Behavior	Grade
**CNN w/ BiRNN - Modular**	92.37	63.16	**92.28**	**79.59**	98.61	79.72
Accuracy	(91.87, 92.37)	(62.78, 63.68)	**(92.01, 92.51)**	**(78.90, 79.63)**	(98.48, 98.70)	(79.26, 80.00)
	**62.14**	27.42	49.89	**32.29**	**73.83**	79.03
Macro F-Score	**(60.26, 62.66)**	(26.16, 27.46)	(46.91, 50.33)	**(30.65, 32.56)**	**(69.56, 78.16)**	(78.66, 80.02)
**CNN w/ BiRNN - End2End**	**92.39**	**63.52**	92.25	78.86	**98.67**	**80.68**
Accuracy	**(91.83, 92.35)**	**(63.12, 64.01)**	(92.17, 92.66)	(78.48, 79.21)	**(98.57, 98.79)**	**(80.67, 81.40)**
	59.21	**27.76**	**50.26**	28.54	72.07	**80.33**
Macro F-Score	(58.96, 91.09)	**(27.40, 28.88)**	**(48.19, 52.68)**	(27.89, 29.66)	(69.63, 78.89)	**(80.11, 81.38)**
**CNN w/ Bi-RNN + CRF - Modular**	**92.26**	**63.03**	**92.29**	**79.27**	98.64	80.66
Accuracy	**(91.97, 92.45)**	**(62.65, 63.59)**	**(92.21, 92.69)**	**(78.62, 79.35)**	(98.50, 98.71)	(80.53, 81.27)
	**64.17**	**32.88**	47.22	33.85	76.22	79.31
Macro F-Score	**(63.89, 66.79)**	**(31.87, 33.49)**	(44.75, 51.84)	(33.88, 35.78)	(74.28, 82.98)	(78.79, 80.20)
**CNN w/ Bi-RNN + CRF - End2End**	91.87	62.45	92.14	78.74	**98.74**	**80.79**
Accuracy	(91.61, 92.11)	(62.18, 63.05)	(91.93, 92.41)	(78.29, 79.03)	**(98.67, 98.88)**	**(80.53, 81.27)**
	60.58	32.87	**49.30**	**34.09**	**80.32**	**80.61**
Macro F-Score	(59.56, 61.79)	(31.99, 33.73)	**(47.69, 52.09)**	**(32.59, 34.21)**	**(78.01, 94.44)**	**(79.95, 81.21)**
**CNN w/ Self-Att - Modular**	**92.60**	**64.40**	92.49	**80.55**	98.73	**82.68**
Accuracy	**(92.32, 92.79)**	**(63.94, 64.84)**	(92.22, 92.67)	**(79.89, 80.66)**	(98.57, 98.78)	**(82.19, 82.87)**
	61.92	30.20	47.52	**35.27**	71.48	**82.55**
Macro F-Score	(60.75, 62.80)	(29.73, 31.20)	(46.36, 50.64)	**(34.02, 35.73)**	(70.06, 79.63)	**(81.59, 82.70)**
**CNN w/ Self-Att - End2End**	92.40	64.21	**92.84**	80.08	**98.87**	81.74
Accuracy	(91.71, 92.22)	(64.13, 65.03)	**(92.36, 92.84)**	(79.55, 80.28)	**(98.75, 98.94)**	(81.17, 81.89)
	**62.90**	**30.77**	**51.35**	33.19	**77.40**	81.32
Macro F-Score	**(60.92, 63.06)**	**(30.37, 31.80)**	**(49.44, 58.75)**	(32.62, 34.37)	**(70.00, 78.23)**	(81.08, 82.13)
**CNN w/ Self-Att + CRF - Modular**	**92.30**	**62.53**	92.15	**78.81**	98.79	**82.08**
Accuracy	**(92.12, 92.60)**	**(62.15, 63.06)**	(91.95, 92.45)	**(78.33, 79.07)**	(98.72, 98.92)	**(82.18, 82.87)**
	**65.41**	**34.46**	49.29	**37.62**	79.22	81.27
Macro F-Score	**(64.67, 67.70)**	**(33.00, 34.62)**	(46.16, 53.53)	**(36.09, 37.81)**	(73.90, 82.66)	(80.79, 82.13)
**CNN w/ Self-Att + CRF - End2End**	92.03	61.38	**91.42**	77.72	**98.82**	81.95
Accuracy	(91.60, 92.10)	(60.89, 61.77)	**(91.06, 91.56)**	(77.14, 77.89)	**(98.71, 99.01)**	(81.27, 82.15)
	63.62	33.99	**51.00**	34.81	**82.10**	**82.07**
Macro F-Score	(62.27, 64.53)	(33.00, 34.66)	**(48.81, 51.51)**	(33.83, 35.56)	**(75.71, 84.27)**	**(80.64, 82.87)**
**CNN w/ RNN - Modular**	**90.60**	61.88	91.43	**76.01**	97.96	**76.49**
Accuracy	**(90.39, 90.91)**	(60.99, 61.92)	(91.20, 91.73)	**(75.55, 76.32)**	(97.81, 98.07)	**(76.15, 76.93)**
	**56.78**	**26.11**	45.73	**28.79**	71.15	**76.80**
Macro F-Score	**(55.68, 58.01)**	**(24.84, 26.10)**	(44.30, 52.28)	**(28.03, 29.77)**	(69.74, 78.59)	**(75.77, 77.22)**
**CNN w/ RNN - End2End**	90.33	**62.16**	**91.56**	75.64	**98.15**	76.35
Accuracy	(90.16, 90.69)	**(61.71, 62.61)**	**(91.19, 91.70)**	(75.72, 76.51)	**(97.92, 98.18)**	(76.01, 76.81)
	55.54	25.06	**46.37**	27.83	**73.44**	76.14
Macro F-Score	(54.05, 56.20)	(24.27, 25.48)	**(45.41, 50.89)**	(26.85, 28.55)	**(72.24, 81.84)**	(74.43, 75.95)
**CNN w/ RNN + CRF - Modular**	**90.82**	**61.50**	91.37	**76.53**	**98.32**	77.23
Accuracy	**(90.56, 91.09)**	**(60.73, 61.63)**	(91.25, 91.78)	**(76.07, 76.85)**	**(98.18, 98.41)**	(76.98, 77,72)
	**60.19**	30.24	**47.65**	32.57	73.05	76.11
Macro F-Score	**(59.01, 61.86)**	(29.71, 31.37)	**(45.04, 48.61)**	(31.21, 33.01)	(69.10, 78.35)	(75.92, 77.29)
**CNN w/ RNN + CRF - End2End**	90.60	61.14	**91.56**	76.50	98.10	**78.46**
Accuracy	(90.40, 91.12)	(60.99, 91.90)	**(91.42, 91.91)**	(76.14, 76.93)	(97.93, 98.18)	**(78.08, 78.86)**
	58.25	**32.79**	47.37	**33.53**	**76.56**	**78.46**
Macro F-Score	(57.40, 59.73)	**(31.73, 33.39)**	(46.51, 54.39)	**(31.89, 33.78)**	**(69.24, 78.65)**	**(77.37, 78.69)**
**CNN w/ Masked SA - Modular**	90.63	**61.72**	**91.35**	76.66	**98.19**	76.88
Accuracy	(90.36, 90.88)	**(60.89, 61.82)**	**(90.90, 91.45)**	(75.92, 76.71)	**(97.91, 98.17)**	(76.33, 77.13)
	59.48	**29.42**	47.44	**30.67**	71.33	76.69
Macro F-Score	(57.80, 60.40)	**(27.78, 30.30)**	(45.02, 49.31)	**(29.53, 31.32)**	(68.46, 77.09)	(75.68, 77.12)
**CNN w/ Masked SA - End2End**	**90.77**	61.53	91.32	**76.79**	98.16	**77.30**
Accuracy	**(90.19, 90.84)**	(60.97, 61.84)	(91.16, 91.66)	**(75.96, 76.78)**	(97.99, 98.25)	**(76.50, 77.46)**
	**60.58**	27.39	**49.26**	30.56	**76.67**	**77.27**
Macro F-Score	**(59.27, 61.76)**	(26.76, 28.30)	**(49.13, 57.61)**	(28.84, 30.97)	**(75.44, 82.82)**	**(76.07, 77.40)**
**CNN w/ M. Self-Att + CRF - Modular**	**91.06**	**62.00**	**91.84**	**77.08**	98.40	**80.54**
Accuracy	**(90.88, 91.41)**	**(61.55, 62.42)**	**(91.38, 91.89)**	**(76.50, 77.27)**	(98.32, 98.54)	**(80.14, 80.88)**
	61.09	30.98	48.14	**33.95**	78.66	**79.92**
Macro F-Score	(60.20, 63.52)	(30.71, 32.37)	(47.10, 51.60)	**(32.86, 34.69)**	(71.72, 80.88)	**(79.09, 80.43)**
**CNN w/ M. Self-Att + CRF - End2End**	90.80	60.60	91.82	75.98	**98.57**	78.56
Accuracy	(90.39, 90.91)	(59.71, 60.72)	(91.55, 92.06)	(75.12, 76.12)	**(98.40, 98.62)**	(78.08, 78.85)
	**61.28**	**31.81**	**50.56**	32.61	**81.22**	78.92
Macro F-Score	**(58.53, 61.03)**	**(30.92, 32.48)**	**(47.99, 52.66)**	(31.34, 33.14)	**(75.20, 81.98)**	(78.20, 79.42)
